# Syntheses and crystal structures of the anhydride 4-oxa­tetra­cyclo­[5.3.2.0^2,6^.0^8,10^]dodec-11-ene-3,5-dione and the related imide 4-(4-bromo­phen­yl)-4-aza­tetra­cyclo­[5.3.2.0^2,6^.0^8,10^]dodec-11-ene-3,5-dione

**DOI:** 10.1107/S2056989020009512

**Published:** 2020-07-17

**Authors:** Andrew Hulsman, Isabel Lorenzana, Theodore Schultz, Breezy Squires, Brock A. Stenfors, Mason Tolonen, Richard J. Staples, Shannon M. Biros, William R. Winchester

**Affiliations:** a Department of Chemistry, Grand Valley State University, 1 Campus Dr., Allendale, MI 49401, USA; b Center for Crystallographic Research, Department of Chemistry, Michigan State University, East Lansing, MI 48824, USA

**Keywords:** crystal structure, C-H⋯O hydrogen bond, C-H⋯π inter­action, lone pair–π inter­action, bi­cyclo­[2.2.2]octene

## Abstract

The syntheses and crystal structures of the two title compounds, C_11_H_10_O_3_ (**I**) and C_17_H_14_BrNO_2_ (**II**), both containing the bi­cyclo­[2.2.2]octene ring system, are reported here [the structure of **I** has been reported previously: White & Goh (2014 ▸). Private Communication (refcode HOKRIK). CCDC, Cambridge, England]. The bond lengths and angles of the bi­cyclo­[2.2.2]octene ring system are similar for both structures. The imide functional group of **II** features carbonyl C=O bond lengths of 1.209 (2) and 1.210 (2) Å, with C—N bond lengths of 1.393 (2) and 1.397 (2) Å. The five-membered imide ring is nearly planar, and it is positioned *exo* relative to the alkene bridgehead carbon atoms of the bi­cyclo­[2.2.2]octene ring system. Non-covalent inter­actions present in the crystal structure of **II** include a number of C—H⋯O inter­actions. The extended structure of **II** also features C—H⋯O hydrogen bonds as well as C—H⋯π and lone pair–π inter­actions, which combine together to create supra­molecular sheets.

## Abstract 2

The Diels–Alder cyclo­addition of cyclo­hepta­triene and maleic anhydride produces the title carb­oxy­lic anhydride; reaction of this anhydride with 4-bromo­phenyl­aniline forms the corresponding tetra­cyclic imide. The anhydride features C—H⋯O hydrogen bonds in the solid state, while the imide also features C—H⋯O hydrogen bonds as well as C—H⋯π and lone pair–π inter­actions.

## Chemical context   

**Figure 1:**
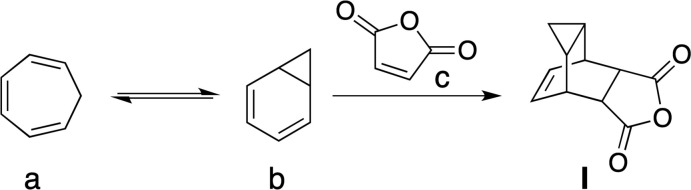
Valence isomerism of cyclo­hepta­triene **a** with norcaradiene **b**, then the Diels–Alder reaction with maleic anhydride **c** to give the title anhydride **I**.

Cyclo­hepta­triene, **a**, exhibits valence isomerism with norcaradiene, **b**, in solution (Fig. 1 ▸). The norcaradiene isomer readily reacts with maleic anhydride, **c**, to form the unique tricyclic anhydride, **I** (White & Goh, 2014 ▸). This reaction has been known since 1939 (Kohler *et al.*, 1939 ▸), but the structure of the major product was not determined until 1953, when it was elucidated that the product contained a cyclo­propane ring (Alder & Jacobs, 1953 ▸). The combination of a rigid tricyclic structure with alkene, anhydride and cyclo­propane functional groups makes this structure inter­esting as a scaffold for drug design because of the ability to specifically place groups in mol­ecular space and thus design mol­ecules to inter­act selectively with protein active sites.
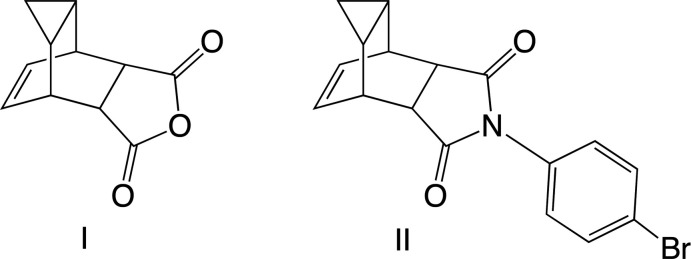


**Figure 2:**
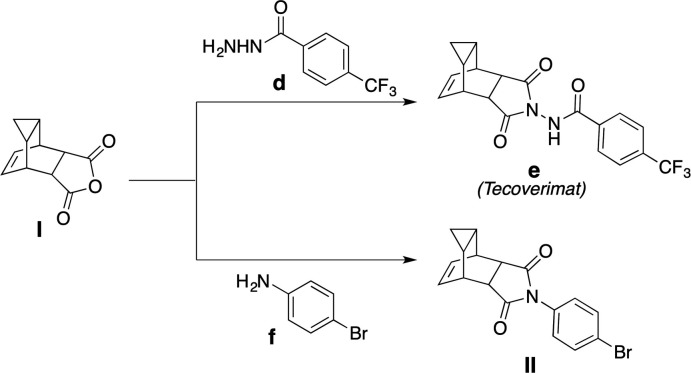
Synthesis of the smallpox anti­viral compound Tecovirimat, and the title imide **II**, which both use anhydride **I** as the starting material.

In a high-throughput screen of 356,000 compounds for activity against vaccinia and cowpox viruses, Bailey *et al.* (2007 ▸) discovered anti­viral activity of imide derivatives related to **I**, including **e** (tecovirimat, C_19_H_15_F_3_N_2_O_3_; Fig. 2 ▸). SAR studies showed that this derivative was the most active of the entire library, and its mode of action was to inhibit extracellular virus formation. Inter­estingly, hydrogenation of the alkene had little effect on the activity of the compound. Tecoviramat has been approved as a treatment for smallpox, and the United States has created a stockpile of two million doses stored at the US Strategic National Stockpile (Hughes, 2019 ▸).

Substituted anilines, such as *p*-bromo­aniline **f**, have also been reacted with the anhydride **I** to form imides that show insecticidal activity (Fig. 2 ▸, Brechbuhler & Petitpierre, 1975 ▸). A wide range of imides were synthesized, including compound **II**, and were shown to protect crops by inhibiting the growth of lepidoptera. Finally, we note that all of these imide derivatives will undergo a *retro*-Diels–Alder cyclo­addition to form cyclo­hepta­triene and a substituted male­imide. Structural investigations have shown that there is an increase in the length of the C—C bonds that are involved in the *retro*-Diels–Alder reaction relative to the other C—C bonds in the mol­ecule (Birney *et al.*, 2002 ▸; Pool *et al.*, 2000 ▸). Herein we report the syntheses and crystal structures of the anhydride **I** and imide **II**. The structure of the anhydride was previously reported as a Private Communication to the CSD (refcode HOKRIK; White & Goh, 2014 ▸).

## Structural commentary   

**Figure 3:**
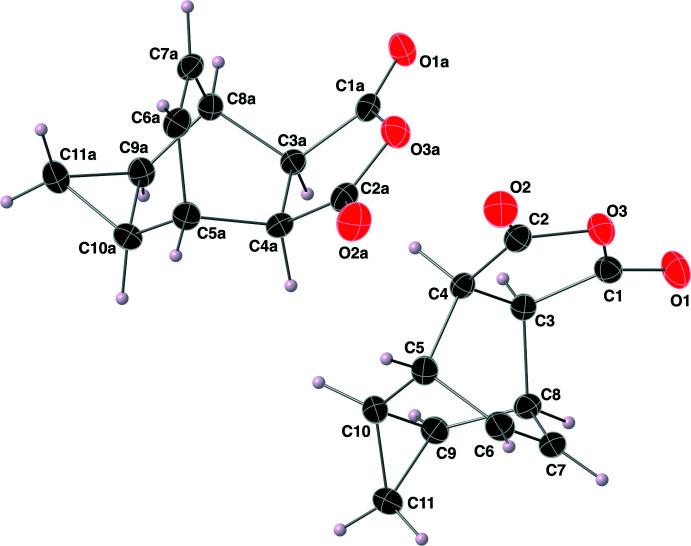
The mol­ecular structure of the anhydride **I**, with the atom-labeling scheme for both crystallographically unique mol­ecules. Displacement ellipsoids are shown at the 40% probability level using standard CPK colors.

The structure of the title anhydride **I** was solved in the monoclinic space group *P*2_1_/*n* with two mol­ecules in the asymmetric unit. The atom labeling scheme (starting with C1 and C1a for the two mol­ecules) is shown in Fig. 3 ▸. This structure is quite similar with respect to the bond lengths and angles described below for the imide **II**. The bond lengths of the carbonyl groups of the anhydride are shorter than the imide, as expected, with C1=O1 = 1.1943 (18), C2=O2 = 1.1904 (17), C1—O3 = 1.3868 (17) and C2—O3 = 1.3978 (16) Å. The corresponding data for the C1a mol­ecule are 1.1913 (17), 1.1871 (18), 1.3855 (17) and 1.3905 (18) Å, respectively. The configurations of the stereogenic centres in the arbitrarily chosen asymmetric mol­ecules are: C3 *S*, C4 *R*, C5 *R*, C8 *S*, C9 *S*, C10 *R* and C3a *R*, C4a *S*, C5a *S*, C8a *R*, C9a *R*, C10a *S*: crystal symmetry generates a racemic mixture in the bulk.

**Figure 4:**
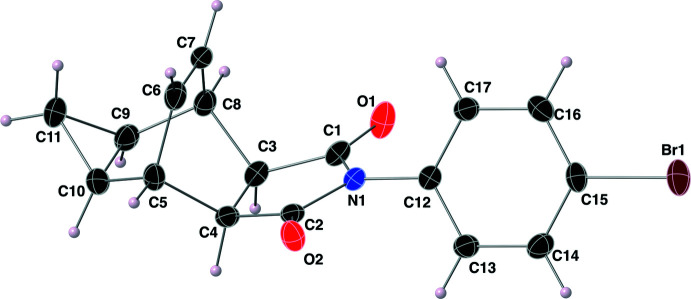
The mol­ecular structure of the imide **II**, with the atom-labeling scheme. Displacement ellipsoids are shown at the 40% probability level using standard CPK colors.

The structure of the imide **II** was solved in the monoclinic space group *P*2_1_/*n*, and its atom labeling scheme is shown in Fig. 4 ▸. The imide functional group of this structure has C=O bond lengths of 1.209 (2) and 1.210 (2) Å, with C—N bond lengths of 1.393 (2) and 1.397 (2) Å. The O—C—N bond angles of the imide functional group are 123.98 (17) and 123.97 (17)°. The aromatic ring, C12–C17, is oriented nearly perpendicular to the plane containing the atoms of the imide functional group with a C1—N1—C12—C17 torsion angle of 65.0 (2)°. The five-membered ring that contains the imide functional group (–C1—N1—C2—C4—C3–) is close to planar with a Cremer–Pople τ value of 2.8 (Cremer & Pople, 1975 ▸). When considering the bi­cyclo­[2.2.2]octene ring system (C3–C10), both C11 and the atoms of the imide functional group are oriented exo relative to the bridgehead alkene carbon atoms C6–C7. The length of the C6=C7 double bond is 1.324 (3) Å, and the cyclo­propyl ring C9–C11 has C—C—C bond angles ranging from 59.89 (13)–60.14 (14)°. The stereogenic centres in the asymmetric mol­ecule of **II** are C3 *R*, C4 *S*, C5 *S*, C8 *R*, C9 *R* and C10 *S*; again, crystal symmetry generates a racemic mixture.

## Supra­molecular features   

**Figure 5:**
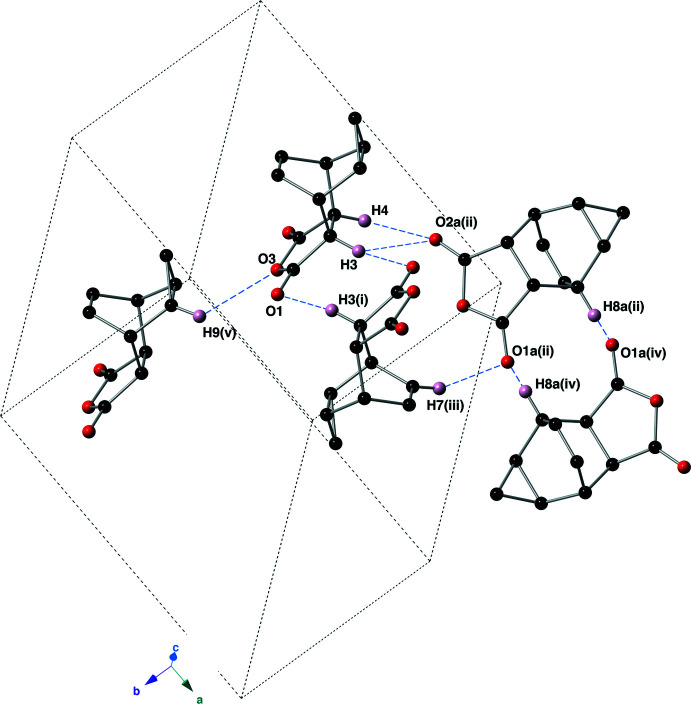
Depiction of the C—H⋯O hydrogen bonds (blue, dashed lines) present in the crystal of anhydride **I**, using a ball-and-stick model. For clarity, only those hydrogen atoms involved in an inter­action are shown. Symmetry codes: (i) −*x* + 2, −*y* + 1, −*z*; (ii) *x* + 1, *y*, *z*; (iii) −*x* + 1, −*y* + 1, −*z*; (iv) −*x* + 1, −*y* + 2, −*z*; (v) *x*, *y* + 1, *z*.

The extended structure of the anhydride **I** is dominated by C—H⋯O hydrogen bonds (Sutor, 1962 ▸, 1963 ▸; Steiner, 1996 ▸) involving both carbonyl groups as acceptors (Table 1 ▸, Fig. 5 ▸). The *D*⋯*A* distances range from 3.1897 (16) to 3.4882 (17) Å with *D*—H⋯*A* angles ranging from 119 to 159°; the C9 bond is likely very weak based on its H⋯*A* distance of 2.73 Å. Combined together, these inter­actions create supra­molecular sheets that lie in the *ab* plane.

**Figure 6:**
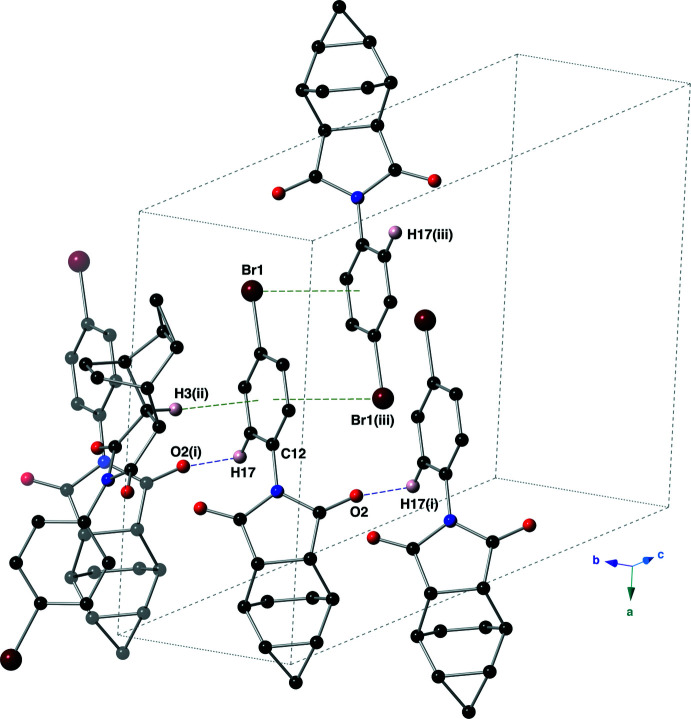
Non-covalent inter­actions present in the crystal of imide **II**, using a ball-and-stick model. Only those hydrogen atoms involved in an inter­action are shown for clarity. C—H⋯O hydrogen bonds are shown with purple, dashed lines, while C—H⋯π and C—Br⋯π inter­actions are shown with green, dashed lines. Symmetry codes: (i) *x*, *y* − 1, *z*; (ii) −*x* + 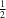, *y* + 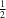, −*z* + 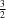.

**Figure 7:**
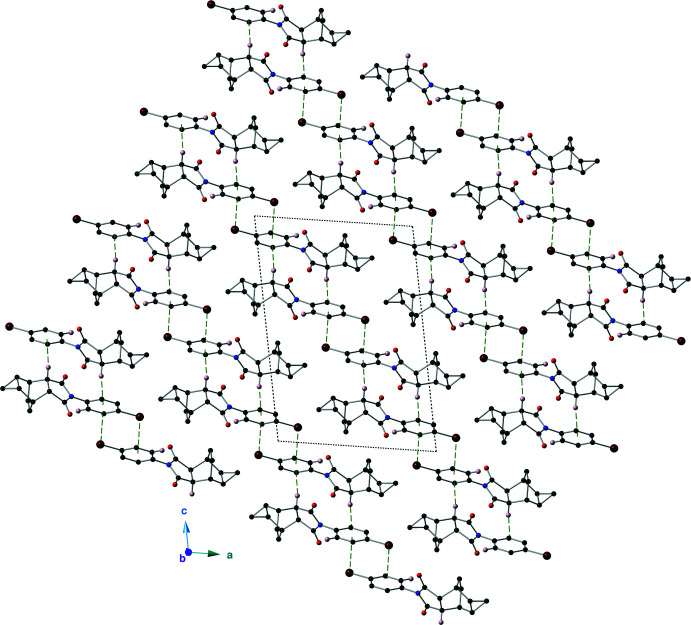
A view of the packing in the crystal of imide **II**, as viewed down the *b* axis. C—H⋯O hydrogen bonds are shown with purple, dashed lines, while C—H⋯π and C—Br⋯π inter­actions are shown with green, dashed lines. For clarity, only those hydrogen atoms involved in a non-covalent inter­action are shown.

In the crystal of the imide **II**, the mol­ecules are linked by C—H⋯O hydrogen bonds as well as C—H⋯π and C—Br⋯π inter­actions (Table 2 ▸, Fig. 6 ▸). The C—H⋯O hydrogen bond is between C17—H17 of the aromatic ring and O2 of an imide carbonyl group. This hydrogen bond has a *D*⋯A distance of 3.175 (2) Å with a *D*—H⋯*A* angle of 139°. The C—H⋯π inter­action is between C3—H3, which is α to the carbonyl group C1(O1), and the aromatic ring C12–C17. This inter­action has a H⋯*Cg* distance of 3.801 (2) Å (where *Cg* is the centroid of the C12–C17 ring), with a C—H⋯*Cg* angle of 165°. The aromatic ring C12–C17 bears an electron-withdrawing bromine atom, and accepts a lone pair(LP)–π inter­action from the bromine atom of a nearby mol­ecule (Mooibroek, *et al.*, 2008 ▸). This LP–π inter­action has a Br⋯*Cg* distance of 3.5854 (8) Å with a C15—Br1⋯*Cg* angle of 87.43 (6)°. Dimers of imide **II** are formed *via* the Br⋯π inter­actions, and these dimers are linked into supra­molecular sheets that lie along (010) by the C—H⋯O and C—H⋯π inter­actions (Fig. 7 ▸).

## Database survey   

The structure of the anhydride **I** has been deposited in the Cambridge Structural Database (CSD, Version 5.41, November, 2019; Groom *et al.*, 2016 ▸) as a Private Communication from White & Goh (2014 ▸, refcode HOKRIK). The acquisition temperature for this data set was 130 K, versus 173 K for the structure reported here. Other than this, the structures are nearly identical. A search of the CSD for structures containing the same bi­cyclo­[2.2.2]octene ring system bearing a cyclic anhydride shows 52 hits (including HOKRIK). Of these, an inter­esting structure is FAXPAV (Coxon *et al.*, 1986 ▸), which bears a very complex fused-ring system in the place of the cyclo­propane ring on anhydride **I**.

A search of the CSD for structures containing a bi­cyclo[2.2.2]octene ring system fused to a cyclic imide resulted in 125 structures related to imide **II**. Structure COZMAH (Wu *et al.*, 2014 ▸) also bears a *p*-bromo­benzene ring bonded to the imide nitro­gen atom, but is derivatized with two esters and an indole ring on the octene portion of the ring system. The structure of tecovirimat (**e**, Fig. 2 ▸) has been deposited as SOKVIY (Bailey *et al.*, 2007 ▸). Finally, structure HARNEV bears two cyclic imide groups on either side of the octene ring system (Song *et al.*, 2012 ▸).

## Synthesis and crystallization   

**Synthesis of the anhydride (I):**

Cyclo­hepta­triene (1.38 g, 15 mmol) and maleic anhydride (1.37 g, 14 mmol) were added to an oven-dried round-bottom flask containing 10 ml of xylene and the mixture was refluxed for 1.5 h. Approximately half of the xylenes were distilled off *via* short-path distillation and the reaction mixture was left to cool at room temperature. The round-bottom flask was fitted with a stopper and left to recrystallize for 48 h to afford large, cream-colored needles. The product was recrystallized once more by dissolving in 8 ml of xylene: after a week at room temperature, the pure product **I** was obtained in the form of large colorless crystals (1.13 g, 40%, m.p. = 372–374 K). ^1^H NMR (400 MHz, chloro­form-*d*) δ 5.88 (*dd*, *J* = 4.8, 3.2 Hz, 2H), 3.46 (*dh*, *J* = 6.6, 2.1 Hz, 2H), 3.23 (*dd*, *J* = 2.1, 1.6 Hz, 2H), 1.17–1.04 (*m*, 4H). ^13^C NMR (101 MHz, chloro­form-*d*) δ 172.45, 128.55, 45.88, 33.65, 9.56, 5.24.

**Synthesis of the imide (II):**

Compound **I** (0.28 g, 1.47 mmol) and *p*-bromo­aniline (0.25 g, 1.45 mmol) were added to a vial containing 5 ml of xylene and the mixture was refluxed for 5 min. The mixture was then cooled to room temperature and left for 5 days in a sealed vial. The precipitate was recrystallized from ethanol solution to yield colorless needle-like crystals of **II** (0.27 g, 52% yield, m.p. = 465–467 K). ^1^H NMR (400 MHz, chloro­form-*d*) δ 7.54 (*d*, *J* = 8.7 Hz, 1H), 7.06 (*d*, *J* = 8.7 Hz, 1H), 5.84 (*dd*, *J* = 4.7, 3.4 Hz, 1H), 3.48 (*s*, 1H), 3.12 (*s*, 1H), 1.14 (*s*, 1H), 0.38–0.21 (*m*, 1H). ^13^C NMR (101 MHz, chloro­form-*d*) δ 177.42, 132.34, 130.88, 128.11, 127.92, 122.47, 45.40, 33.90, 9.97, 4.80.

## Refinement   

Crystal data, data collection and structure refinement details are summarized in Table 3 ▸. For both structures, hydrogen atoms bonded to carbon atoms were placed in calculated positions and refined to ride on their parent atoms: C—H = 0.95–1.00 Å with *U*_iso_(H) = 1.2*U*_eq_(C).

## Supplementary Material

Crystal structure: contains datablock(s) global, II, I. DOI: 10.1107/S2056989020009512/hb7931sup1.cif

Structure factors: contains datablock(s) I. DOI: 10.1107/S2056989020009512/hb7931Isup3.hkl

Structure factors: contains datablock(s) II. DOI: 10.1107/S2056989020009512/hb7931IIsup4.hkl

Supporting information file. DOI: 10.1107/S2056989020009512/hb7931Isup4.cml

Supporting information file. DOI: 10.1107/S2056989020009512/hb7931IIsup5.cml

CCDC references: 2015807, 2015806

Additional supporting information:  crystallographic information; 3D view; checkCIF report

## Figures and Tables

**Table 1:** Hydrogen-bond geometry (Å, °) for **I** [Chem scheme1]

*D*—H⋯*A*	*D*—H	H⋯*A*	*D*⋯*A*	*D*—H⋯*A*
C3—H3⋯O1^i^	1.00	2.64	3.4882 (17)	143
C3—H3⋯O2*A* ^ii^	1.00	2.54	3.2487 (17)	128
C4—H4⋯O2*A* ^ii^	1.00	2.43	3.1897 (16)	133
C7—H7⋯O1*A* ^iii^	0.95	2.56	3.4652 (18)	159
C8*A*—H8*A*⋯O1*A* ^iv^	1.00	2.59	3.2521 (17)	123
C9—H9⋯O3^v^	1.00	2.73	3.3409 (17)	119

Symmetry codes: (i) 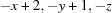; (ii) 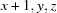; (iii) 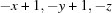; (iv) 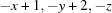; (v) 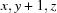.

**Table 2:** Hydrogen-bond geometry (Å, °) for **II** [Chem scheme1] *Cg*1 is the centroid of the C12–C17 ring.

*D*—H⋯*A*	*D*—H	H⋯*A*	*D*⋯*A*	*D*—H⋯*A*
C17—H17⋯O2^i^	0.95	2.40	3.175 (2)	139
C3—H3⋯*Cg*1^ii^	1.00	2.83	3.801 (2)	165

Symmetry codes: (i) 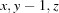; (ii) 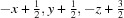.

**Table 3:** Experimental details

	**I**	**II**
Crystal data		
Chemical formula	C_11_H_10_O_3_	C_17_H_14_BrNO_2_
*M*_r_	190.19	344.20
Crystal system, space group	Monoclinic, *P*2_1_/*n*	Monoclinic, *P*2_1_/*n*
Temperature (K)	173	173
*a*, *b*, *c* (Å)	11.3538 (3), 7.4062 (2), 20.5398 (5)	12.49907 (16), 6.41302 (8), 17.8772 (2)
β (°)	92.6226 (15)	99.8083 (6)
*V* (Å^3^)	1725.35 (8)	1412.04 (3)
*Z*	8	4
Radiation type	Cu *K*α	Cu *K*α
μ (mm^−1^)	0.88	4.00
Crystal size (mm)	0.53 × 0.32 × 0.22	0.42 × 0.12 × 0.04

Data collection		
Diffractometer	Bruker APEXII CCD	Bruker APEXII CCD
Absorption correction	Multi-scan (*SADABS*; Bruker, 2013 ▸)	Multi-scan (*SADABS*; Bruker, 2013 ▸)
*T*_min_, *T*_max_	0.675, 0.754	0.578, 0.753
No. of measured, independent and observed [*I* > 2σ(*I*)] reflections	13466, 3353, 3070	23789, 2683, 2441
*R*_int_	0.028	0.039
(sin θ/λ)_max_ (Å^−1^)	0.618	0.610

Refinement		
*R*[*F* ^2^ > 2σ(*F* ^2^)], *wR*(*F* ^2^), *S*	0.045, 0.116, 1.06	0.027, 0.072, 1.04
No. of reflections	3353	2683
No. of parameters	253	190
H-atom treatment	H-atom parameters constrained	H-atom parameters constrained
Δρ_max_, Δρ_min_ (e Å^−3^)	0.22, −0.40	0.56, −0.48

Computer programs: *APEX2* and *SAINT* (Bruker, 2013 ▸), *SHELXS* (Sheldrick, 2008 ▸), *SHELXL* (Sheldrick, 2015 ▸), *OLEX2* (Dolomanov *et al.*, 2009 ▸; Bourhis *et al.*, 2015 ▸) and *CrystalMaker* (Palmer, 2007 ▸).

## supplementary crystallographic information

### 4-Oxatetracyclo[5.3.2.02,6.08,10]dodec-11-ene-3,5-dione (I) Crystal data

**Table:**

C_11_H_10_O_3_	*F*(000) = 800
*M**_r_* = 190.19	*D*_x_ = 1.464 Mg m^−^^3^
Monoclinic, *P*2_1_/*n*	Cu *K*α radiation, λ = 1.54178 Å
*a* = 11.3538 (3) Å	Cell parameters from 8934 reflections
*b* = 7.4062 (2) Å	θ = 3.9–72.4°
*c* = 20.5398 (5) Å	µ = 0.88 mm^−^^1^
β = 92.6226 (15)°	*T* = 173 K
*V* = 1725.35 (8) Å^3^	Chunk, colourless
*Z* = 8	0.53 × 0.32 × 0.22 mm

### 4-Oxatetracyclo[5.3.2.02,6.08,10]dodec-11-ene-3,5-dione (I) Data collection

**Table:**

Bruker APEXII CCD diffractometer	3070 reflections with *I* > 2σ(*I*)
φ and ω scans	*R*_int_ = 0.028
Absorption correction: multi-scan (SADABS; Bruker, 2013)	θ_max_ = 72.4°, θ_min_ = 4.3°
*T*_min_ = 0.675, *T*_max_ = 0.754	*h* = −14→13
13466 measured reflections	*k* = −9→9
3353 independent reflections	*l* = −25→25

### 4-Oxatetracyclo[5.3.2.02,6.08,10]dodec-11-ene-3,5-dione (I) Refinement

**Table:**

Refinement on *F*^2^	Primary atom site location: structure-invariant direct methods
Least-squares matrix: full	Hydrogen site location: inferred from neighbouring sites
*R*[*F*^2^ > 2σ(*F*^2^)] = 0.045	H-atom parameters constrained
*wR*(*F*^2^) = 0.116	*w* = 1/[σ^2^(*F*_o_^2^) + (0.0673*P*)^2^ + 0.5724*P*] where *P* = (*F*_o_^2^ + 2*F*_c_^2^)/3
*S* = 1.06	(Δ/σ)_max_ < 0.001
3353 reflections	Δρ_max_ = 0.22 e Å^−^^3^
253 parameters	Δρ_min_ = −0.40 e Å^−^^3^
0 restraints	

### 4-Oxatetracyclo[5.3.2.02,6.08,10]dodec-11-ene-3,5-dione (I) Special details

**Table:**

Geometry. All esds (except the esd in the dihedral angle between two l.s. planes) are estimated using the full covariance matrix. The cell esds are taken into account individually in the estimation of esds in distances, angles and torsion angles; correlations between esds in cell parameters are only used when they are defined by crystal symmetry. An approximate (isotropic) treatment of cell esds is used for estimating esds involving l.s. planes.

### 4-Oxatetracyclo[5.3.2.02,6.08,10]dodec-11-ene-3,5-dione (I) Fractional atomic coordinates and isotropic or equivalent isotropic displacement parameters (Å^2^)

**Table:**

	*x*	*y*	*z*	*U*_iso_*/*U*_eq_	
O1	0.92952 (11)	0.22515 (15)	0.01539 (5)	0.0378 (3)	
O2	0.91944 (10)	0.21601 (15)	0.23113 (5)	0.0354 (3)	
O3	0.92868 (9)	0.18344 (13)	0.12312 (5)	0.0287 (2)	
C1	0.92625 (12)	0.29232 (19)	0.06814 (7)	0.0249 (3)	
C2	0.92145 (12)	0.28818 (19)	0.17941 (7)	0.0243 (3)	
C3	0.92092 (11)	0.48779 (18)	0.08716 (6)	0.0212 (3)	
H3	0.9931	0.5521	0.0734	0.025*	
C4	0.91806 (11)	0.48510 (18)	0.16185 (6)	0.0206 (3)	
H4	0.9892	0.5477	0.1814	0.025*	
C5	0.80361 (12)	0.57992 (19)	0.18403 (6)	0.0240 (3)	
H5	0.7976	0.5761	0.2324	0.029*	
C6	0.70138 (12)	0.4864 (2)	0.14922 (7)	0.0284 (3)	
H6	0.6400	0.4300	0.1717	0.034*	
C7	0.70364 (12)	0.4889 (2)	0.08459 (8)	0.0288 (3)	
H7	0.6438	0.4348	0.0573	0.035*	
C8	0.80824 (12)	0.58391 (19)	0.05799 (6)	0.0245 (3)	
H8	0.8054	0.5833	0.0093	0.029*	
C9	0.81906 (12)	0.77608 (19)	0.08545 (7)	0.0259 (3)	
H9	0.8782	0.8575	0.0659	0.031*	
C10	0.81589 (12)	0.77348 (19)	0.15890 (7)	0.0255 (3)	
H10	0.8732	0.8536	0.1835	0.031*	
C11	0.71901 (13)	0.8646 (2)	0.11912 (8)	0.0317 (3)	
H11C	0.7163	0.9982	0.1197	0.038*	
H11D	0.6410	0.8046	0.1159	0.038*	
O1A	0.45014 (10)	0.77462 (14)	0.02985 (5)	0.0328 (3)	
O2A	0.12278 (10)	0.77025 (15)	0.13936 (6)	0.0386 (3)	
O3A	0.28206 (9)	0.73501 (13)	0.08039 (5)	0.0313 (3)	
C1A	0.36811 (12)	0.84253 (19)	0.05447 (6)	0.0241 (3)	
C2A	0.19782 (12)	0.8398 (2)	0.10974 (7)	0.0270 (3)	
C3A	0.34016 (11)	1.03897 (17)	0.06434 (6)	0.0206 (3)	
H3A	0.3307	1.1025	0.0215	0.025*	
C4A	0.22267 (11)	1.03737 (18)	0.09904 (6)	0.0219 (3)	
H4A	0.1585	1.0939	0.0710	0.026*	
C5A	0.23788 (12)	1.13775 (19)	0.16575 (6)	0.0239 (3)	
H5A	0.1634	1.1373	0.1899	0.029*	
C6A	0.33646 (12)	1.04165 (19)	0.20315 (6)	0.0254 (3)	
H6A	0.3267	0.9881	0.2446	0.031*	
C7A	0.43832 (12)	1.03759 (19)	0.17356 (6)	0.0240 (3)	
H7A	0.5066	0.9791	0.1919	0.029*	
C8A	0.43686 (11)	1.13289 (18)	0.10893 (6)	0.0216 (3)	
H8A	0.5158	1.1289	0.0893	0.026*	
C9A	0.39235 (12)	1.32691 (19)	0.11555 (7)	0.0250 (3)	
H9A	0.4001	1.4080	0.0771	0.030*	
C10A	0.27558 (12)	1.32973 (19)	0.14820 (7)	0.0260 (3)	
H10A	0.2131	1.4120	0.1293	0.031*	
C11A	0.38257 (13)	1.4167 (2)	0.18102 (7)	0.0307 (3)	
H11A	0.4195	1.3554	0.2196	0.037*	
H11B	0.3854	1.5503	0.1826	0.037*	

### 4-Oxatetracyclo[5.3.2.02,6.08,10]dodec-11-ene-3,5-dione (I) Atomic displacement parameters (Å^2^)

**Table:**

	*U*^11^	*U*^22^	*U*^33^	*U*^12^	*U*^13^	*U*^23^
O1	0.0524 (7)	0.0336 (6)	0.0279 (6)	0.0065 (5)	0.0060 (5)	−0.0079 (4)
O2	0.0469 (7)	0.0310 (6)	0.0285 (5)	−0.0024 (5)	0.0018 (5)	0.0078 (4)
O3	0.0367 (6)	0.0208 (5)	0.0286 (5)	0.0005 (4)	0.0026 (4)	−0.0004 (4)
C1	0.0235 (6)	0.0260 (7)	0.0255 (7)	0.0018 (5)	0.0032 (5)	−0.0010 (5)
C2	0.0223 (6)	0.0261 (7)	0.0246 (7)	−0.0028 (5)	0.0009 (5)	0.0001 (5)
C3	0.0198 (6)	0.0226 (6)	0.0212 (6)	−0.0012 (5)	0.0027 (5)	−0.0005 (5)
C4	0.0186 (6)	0.0226 (6)	0.0205 (6)	−0.0031 (5)	0.0007 (5)	−0.0005 (5)
C5	0.0220 (6)	0.0265 (7)	0.0238 (6)	−0.0006 (5)	0.0050 (5)	−0.0031 (5)
C6	0.0192 (6)	0.0274 (7)	0.0391 (8)	−0.0028 (5)	0.0066 (6)	−0.0035 (6)
C7	0.0210 (7)	0.0274 (7)	0.0375 (8)	−0.0006 (5)	−0.0039 (6)	−0.0077 (6)
C8	0.0253 (7)	0.0258 (7)	0.0220 (6)	0.0028 (5)	−0.0025 (5)	−0.0014 (5)
C9	0.0263 (7)	0.0232 (7)	0.0281 (7)	0.0024 (5)	−0.0008 (5)	0.0013 (5)
C10	0.0245 (7)	0.0241 (7)	0.0279 (7)	0.0000 (5)	0.0004 (5)	−0.0051 (5)
C11	0.0281 (7)	0.0269 (7)	0.0398 (8)	0.0063 (6)	−0.0011 (6)	−0.0046 (6)
O1A	0.0345 (6)	0.0312 (5)	0.0329 (5)	0.0046 (4)	0.0041 (4)	−0.0083 (4)
O2A	0.0344 (6)	0.0365 (6)	0.0454 (7)	−0.0156 (5)	0.0066 (5)	0.0019 (5)
O3A	0.0321 (6)	0.0218 (5)	0.0399 (6)	−0.0027 (4)	0.0022 (4)	−0.0014 (4)
C1A	0.0268 (7)	0.0251 (7)	0.0201 (6)	−0.0015 (5)	−0.0031 (5)	−0.0024 (5)
C2A	0.0242 (7)	0.0278 (7)	0.0287 (7)	−0.0053 (5)	−0.0024 (5)	−0.0007 (6)
C3A	0.0226 (6)	0.0217 (6)	0.0175 (6)	0.0003 (5)	0.0003 (5)	0.0014 (5)
C4A	0.0188 (6)	0.0245 (7)	0.0221 (6)	−0.0008 (5)	−0.0009 (5)	0.0019 (5)
C5A	0.0211 (6)	0.0275 (7)	0.0236 (6)	−0.0012 (5)	0.0050 (5)	−0.0008 (5)
C6A	0.0284 (7)	0.0299 (7)	0.0180 (6)	−0.0031 (6)	0.0009 (5)	0.0014 (5)
C7A	0.0237 (6)	0.0266 (7)	0.0215 (6)	0.0004 (5)	−0.0031 (5)	−0.0011 (5)
C8A	0.0194 (6)	0.0239 (7)	0.0218 (6)	−0.0021 (5)	0.0031 (5)	−0.0018 (5)
C9A	0.0260 (7)	0.0225 (7)	0.0267 (7)	−0.0031 (5)	0.0046 (5)	−0.0005 (5)
C10A	0.0261 (7)	0.0239 (7)	0.0282 (7)	0.0029 (5)	0.0043 (5)	−0.0021 (5)
C11A	0.0328 (8)	0.0265 (7)	0.0330 (8)	−0.0023 (6)	0.0037 (6)	−0.0070 (6)

### 4-Oxatetracyclo[5.3.2.02,6.08,10]dodec-11-ene-3,5-dione (I) Geometric parameters (Å, º)

**Table:**

O1—C1	1.1943 (18)	O1A—C1A	1.1913 (17)
O2—C2	1.1904 (17)	O2A—C2A	1.1871 (18)
O3—C1	1.3868 (17)	O3A—C1A	1.3855 (17)
O3—C2	1.3978 (16)	O3A—C2A	1.3905 (18)
C1—C3	1.5014 (18)	C1A—C3A	1.5048 (18)
C2—C4	1.5024 (18)	C2A—C4A	1.5080 (19)
C3—H3	1.0000	C3A—H3A	1.0000
C3—C4	1.5360 (17)	C3A—C4A	1.5409 (17)
C3—C8	1.5596 (18)	C3A—C8A	1.5607 (17)
C4—H4	1.0000	C4A—H4A	1.0000
C4—C5	1.5631 (17)	C4A—C5A	1.5616 (18)
C5—H5	1.0000	C5A—H5A	1.0000
C5—C6	1.5040 (19)	C5A—C6A	1.5068 (19)
C5—C10	1.5321 (19)	C5A—C10A	1.5326 (19)
C6—H6	0.9500	C6A—H6A	0.9500
C6—C7	1.329 (2)	C6A—C7A	1.3312 (19)
C7—H7	0.9500	C7A—H7A	0.9500
C7—C8	1.504 (2)	C7A—C8A	1.5028 (18)
C8—H8	1.0000	C8A—H8A	1.0000
C8—C9	1.5337 (19)	C8A—C9A	1.5313 (19)
C9—H9	1.0000	C9A—H9A	1.0000
C9—C10	1.5110 (19)	C9A—C10A	1.5131 (18)
C9—C11	1.5066 (19)	C9A—C11A	1.5091 (19)
C10—H10	1.0000	C10A—H10A	1.0000
C10—C11	1.500 (2)	C10A—C11A	1.507 (2)
C11—H11C	0.9900	C11A—H11A	0.9900
C11—H11D	0.9900	C11A—H11B	0.9900
			
C1—O3—C2	110.55 (11)	C1A—O3A—C2A	110.90 (11)
O1—C1—O3	119.75 (13)	O1A—C1A—O3A	119.95 (13)
O1—C1—C3	129.86 (13)	O1A—C1A—C3A	129.73 (13)
O3—C1—C3	110.39 (11)	O3A—C1A—C3A	110.31 (11)
O2—C2—O3	119.55 (13)	O2A—C2A—O3A	120.26 (14)
O2—C2—C4	130.45 (13)	O2A—C2A—C4A	129.74 (14)
O3—C2—C4	110.00 (11)	O3A—C2A—C4A	109.97 (11)
C1—C3—H3	110.1	C1A—C3A—H3A	110.6
C1—C3—C4	104.49 (10)	C1A—C3A—C4A	104.31 (11)
C1—C3—C8	112.48 (11)	C1A—C3A—C8A	111.28 (11)
C4—C3—H3	110.1	C4A—C3A—H3A	110.6
C4—C3—C8	109.58 (10)	C4A—C3A—C8A	109.43 (10)
C8—C3—H3	110.1	C8A—C3A—H3A	110.6
C2—C4—C3	104.53 (10)	C2A—C4A—C3A	104.27 (11)
C2—C4—H4	110.0	C2A—C4A—H4A	110.7
C2—C4—C5	112.25 (11)	C2A—C4A—C5A	110.36 (11)
C3—C4—H4	110.0	C3A—C4A—H4A	110.7
C3—C4—C5	109.95 (10)	C3A—C4A—C5A	109.80 (10)
C5—C4—H4	110.0	C5A—C4A—H4A	110.7
C4—C5—H5	111.9	C4A—C5A—H5A	111.8
C6—C5—C4	106.76 (11)	C6A—C5A—C4A	105.79 (10)
C6—C5—H5	111.9	C6A—C5A—H5A	111.8
C6—C5—C10	110.54 (12)	C6A—C5A—C10A	110.44 (11)
C10—C5—C4	103.44 (10)	C10A—C5A—C4A	104.84 (10)
C10—C5—H5	111.9	C10A—C5A—H5A	111.8
C5—C6—H6	122.6	C5A—C6A—H6A	122.6
C7—C6—C5	114.75 (12)	C7A—C6A—C5A	114.74 (12)
C7—C6—H6	122.6	C7A—C6A—H6A	122.6
C6—C7—H7	122.6	C6A—C7A—H7A	122.6
C6—C7—C8	114.89 (12)	C6A—C7A—C8A	114.71 (12)
C8—C7—H7	122.6	C8A—C7A—H7A	122.6
C3—C8—H8	111.8	C3A—C8A—H8A	111.7
C7—C8—C3	107.11 (11)	C7A—C8A—C3A	106.74 (10)
C7—C8—H8	111.8	C7A—C8A—H8A	111.7
C7—C8—C9	110.60 (11)	C7A—C8A—C9A	110.65 (11)
C9—C8—C3	103.41 (10)	C9A—C8A—C3A	104.13 (10)
C9—C8—H8	111.8	C9A—C8A—H8A	111.7
C8—C9—H9	117.1	C8A—C9A—H9A	116.9
C10—C9—C8	110.50 (11)	C10A—C9A—C8A	110.59 (11)
C10—C9—H9	117.1	C10A—C9A—H9A	116.9
C11—C9—C8	121.54 (12)	C11A—C9A—C8A	122.06 (12)
C11—C9—H9	117.1	C11A—C9A—H9A	116.9
C11—C9—C10	59.62 (9)	C11A—C9A—C10A	59.82 (9)
C5—C10—H10	116.9	C5A—C10A—H10A	117.1
C9—C10—C5	110.79 (11)	C9A—C10A—C5A	110.56 (11)
C9—C10—H10	116.9	C9A—C10A—H10A	117.1
C11—C10—C5	121.90 (12)	C11A—C10A—C5A	121.31 (12)
C11—C10—C9	60.05 (9)	C11A—C10A—C9A	59.96 (9)
C11—C10—H10	116.9	C11A—C10A—H10A	117.1
C9—C11—H11C	117.7	C9A—C11A—H11A	117.7
C9—C11—H11D	117.7	C9A—C11A—H11B	117.7
C10—C11—C9	60.33 (9)	C10A—C11A—C9A	60.22 (9)
C10—C11—H11C	117.7	C10A—C11A—H11A	117.7
C10—C11—H11D	117.7	C10A—C11A—H11B	117.7
H11C—C11—H11D	114.9	H11A—C11A—H11B	114.9

### 4-Oxatetracyclo[5.3.2.02,6.08,10]dodec-11-ene-3,5-dione (I) Hydrogen-bond geometry (Å, º)

**Table:**

*D*—H···*A*	*D*—H	H···*A*	*D*···*A*	*D*—H···*A*
C3—H3···O1^i^	1.00	2.64	3.4882 (17)	143
C3—H3···O2*A*^ii^	1.00	2.54	3.2487 (17)	128
C4—H4···O2*A*^ii^	1.00	2.43	3.1897 (16)	133
C7—H7···O1*A*^iii^	0.95	2.56	3.4652 (18)	159
C8*A*—H8*A*···O1*A*^iv^	1.00	2.59	3.2521 (17)	123
C9—H9···O3^v^	1.00	2.73	3.3409 (17)	119

Symmetry codes: (i) −*x*+2, −*y*+1, −*z*; (ii) *x*+1, *y*, *z*; (iii) −*x*+1, −*y*+1, −*z*; (iv) −*x*+1, −*y*+2, −*z*; (v) *x*, *y*+1, *z*.

### 4-(4-Bromophenyl)-4-azatetracyclo[5.3.2.02,6.08,10]dodec-11-ene-3,5-dione (II) Crystal data

**Table:**

C_17_H_14_BrNO_2_	*F*(000) = 696
*M**_r_* = 344.20	*D*_x_ = 1.619 Mg m^−^^3^
Monoclinic, *P*2_1_/*n*	Cu *K*α radiation, λ = 1.54178 Å
*a* = 12.49907 (16) Å	Cell parameters from 9902 reflections
*b* = 6.41302 (8) Å	θ = 4.7–70.1°
*c* = 17.8772 (2) Å	µ = 4.00 mm^−^^1^
β = 99.8083 (6)°	*T* = 173 K
*V* = 1412.04 (3) Å^3^	Needle, colourless
*Z* = 4	0.42 × 0.12 × 0.04 mm

### 4-(4-Bromophenyl)-4-azatetracyclo[5.3.2.02,6.08,10]dodec-11-ene-3,5-dione (II) Data collection

**Table:**

Bruker APEXII CCD diffractometer	2441 reflections with *I* > 2σ(*I*)
φ and ω scans	*R*_int_ = 0.039
Absorption correction: multi-scan (SADABS; Bruker, 2013)	θ_max_ = 70.2°, θ_min_ = 4.0°
*T*_min_ = 0.578, *T*_max_ = 0.753	*h* = −15→15
23789 measured reflections	*k* = −7→7
2683 independent reflections	*l* = −21→21

### 4-(4-Bromophenyl)-4-azatetracyclo[5.3.2.02,6.08,10]dodec-11-ene-3,5-dione (II) Refinement

**Table:**

Refinement on *F*^2^	Primary atom site location: structure-invariant direct methods
Least-squares matrix: full	Hydrogen site location: inferred from neighbouring sites
*R*[*F*^2^ > 2σ(*F*^2^)] = 0.027	H-atom parameters constrained
*wR*(*F*^2^) = 0.072	*w* = 1/[σ^2^(*F*_o_^2^) + (0.033*P*)^2^ + 1.1866*P*] where *P* = (*F*_o_^2^ + 2*F*_c_^2^)/3
*S* = 1.04	(Δ/σ)_max_ = 0.003
2683 reflections	Δρ_max_ = 0.56 e Å^−^^3^
190 parameters	Δρ_min_ = −0.48 e Å^−^^3^
0 restraints	

### 4-(4-Bromophenyl)-4-azatetracyclo[5.3.2.02,6.08,10]dodec-11-ene-3,5-dione (II) Special details

**Table:**

Geometry. All esds (except the esd in the dihedral angle between two l.s. planes) are estimated using the full covariance matrix. The cell esds are taken into account individually in the estimation of esds in distances, angles and torsion angles; correlations between esds in cell parameters are only used when they are defined by crystal symmetry. An approximate (isotropic) treatment of cell esds is used for estimating esds involving l.s. planes.

### 4-(4-Bromophenyl)-4-azatetracyclo[5.3.2.02,6.08,10]dodec-11-ene-3,5-dione (II) Fractional atomic coordinates and isotropic or equivalent isotropic displacement parameters (Å^2^)

**Table:**

	*x*	*y*	*z*	*U*_iso_*/*U*_eq_	
Br1	0.63472 (2)	−0.24792 (4)	0.56752 (2)	0.03728 (10)	
O1	0.18672 (12)	−0.1467 (3)	0.72460 (9)	0.0360 (4)	
O2	0.19184 (11)	0.3903 (2)	0.55825 (8)	0.0307 (3)	
N1	0.21210 (12)	0.1061 (2)	0.63812 (8)	0.0204 (3)	
C1	0.16094 (14)	0.0182 (3)	0.69397 (10)	0.0237 (4)	
C2	0.16488 (15)	0.2941 (3)	0.61018 (11)	0.0218 (4)	
C3	0.07071 (14)	0.1617 (3)	0.70770 (10)	0.0233 (4)	
H3	0.0846	0.2102	0.7616	0.028*	
C4	0.07607 (14)	0.3487 (3)	0.65452 (10)	0.0229 (4)	
H4	0.0962	0.4777	0.6851	0.028*	
C5	−0.03628 (15)	0.3789 (3)	0.60269 (11)	0.0257 (4)	
H5	−0.0355	0.4979	0.5667	0.031*	
C6	−0.06246 (15)	0.1766 (3)	0.56169 (11)	0.0288 (4)	
H6	−0.0753	0.1670	0.5079	0.035*	
C7	−0.06639 (15)	0.0122 (3)	0.60590 (12)	0.0280 (4)	
H7	−0.0819	−0.1243	0.5864	0.034*	
C8	−0.04426 (14)	0.0588 (3)	0.68951 (11)	0.0258 (4)	
H8	−0.0490	−0.0694	0.7206	0.031*	
C9	−0.12081 (16)	0.2311 (3)	0.70832 (12)	0.0285 (4)	
H9	−0.1212	0.2589	0.7633	0.034*	
C10	−0.11491 (16)	0.4179 (3)	0.65832 (12)	0.0300 (4)	
H10	−0.1116	0.5579	0.6832	0.036*	
C11	−0.21970 (16)	0.2988 (4)	0.65312 (14)	0.0348 (5)	
H11A	−0.2427	0.2114	0.6076	0.042*	
H11B	−0.2797	0.3652	0.6741	0.042*	
C12	0.31028 (14)	0.0235 (3)	0.61886 (10)	0.0209 (4)	
C13	0.40447 (15)	0.1405 (3)	0.63508 (11)	0.0262 (4)	
H13	0.4030	0.2752	0.6569	0.031*	
C14	0.50106 (16)	0.0594 (3)	0.61918 (11)	0.0296 (4)	
H14	0.5662	0.1383	0.6299	0.035*	
C15	0.50157 (15)	−0.1369 (3)	0.58771 (10)	0.0254 (4)	
C16	0.40808 (18)	−0.2541 (3)	0.57108 (11)	0.0284 (4)	
H16	0.4098	−0.3887	0.5491	0.034*	
C17	0.31120 (15)	−0.1728 (3)	0.58690 (11)	0.0253 (4)	
H17	0.2461	−0.2516	0.5758	0.030*	

### 4-(4-Bromophenyl)-4-azatetracyclo[5.3.2.02,6.08,10]dodec-11-ene-3,5-dione (II) Atomic displacement parameters (Å^2^)

**Table:**

	*U*^11^	*U*^22^	*U*^33^	*U*^12^	*U*^13^	*U*^23^
Br1	0.03088 (14)	0.04690 (17)	0.03609 (15)	0.01410 (9)	0.01146 (10)	−0.00083 (10)
O1	0.0294 (7)	0.0422 (9)	0.0382 (8)	0.0087 (6)	0.0107 (6)	0.0211 (7)
O2	0.0312 (7)	0.0263 (7)	0.0371 (8)	0.0002 (6)	0.0131 (6)	0.0095 (6)
N1	0.0175 (7)	0.0233 (8)	0.0205 (7)	−0.0015 (6)	0.0031 (6)	0.0013 (6)
C1	0.0184 (8)	0.0322 (10)	0.0197 (8)	−0.0015 (7)	0.0008 (7)	0.0046 (8)
C2	0.0201 (8)	0.0194 (8)	0.0252 (9)	−0.0045 (7)	0.0019 (7)	−0.0007 (7)
C3	0.0197 (8)	0.0319 (10)	0.0184 (8)	−0.0016 (7)	0.0034 (7)	0.0010 (8)
C4	0.0221 (9)	0.0213 (9)	0.0257 (9)	−0.0033 (7)	0.0049 (7)	−0.0033 (7)
C5	0.0235 (9)	0.0243 (9)	0.0295 (9)	0.0043 (7)	0.0049 (7)	0.0054 (8)
C6	0.0211 (9)	0.0397 (11)	0.0239 (9)	0.0038 (8)	−0.0010 (7)	−0.0059 (9)
C7	0.0180 (8)	0.0260 (10)	0.0394 (11)	−0.0015 (7)	0.0028 (8)	−0.0097 (9)
C8	0.0191 (9)	0.0276 (10)	0.0315 (10)	−0.0018 (7)	0.0063 (7)	0.0049 (8)
C9	0.0210 (9)	0.0346 (11)	0.0312 (10)	−0.0008 (8)	0.0083 (8)	−0.0033 (8)
C10	0.0241 (9)	0.0272 (10)	0.0400 (11)	0.0023 (8)	0.0093 (8)	−0.0043 (9)
C11	0.0203 (10)	0.0382 (11)	0.0465 (12)	0.0036 (9)	0.0074 (9)	−0.0021 (10)
C12	0.0201 (8)	0.0247 (9)	0.0180 (8)	0.0000 (7)	0.0037 (7)	0.0022 (7)
C13	0.0248 (9)	0.0266 (10)	0.0284 (9)	−0.0035 (8)	0.0080 (7)	−0.0061 (8)
C14	0.0215 (9)	0.0362 (11)	0.0322 (10)	−0.0042 (8)	0.0082 (8)	−0.0057 (9)
C15	0.0245 (9)	0.0315 (10)	0.0213 (8)	0.0084 (8)	0.0071 (7)	0.0025 (8)
C16	0.0358 (11)	0.0239 (10)	0.0253 (9)	0.0045 (8)	0.0042 (8)	−0.0017 (8)
C17	0.0244 (9)	0.0246 (9)	0.0259 (9)	−0.0023 (7)	0.0011 (7)	0.0013 (8)

### 4-(4-Bromophenyl)-4-azatetracyclo[5.3.2.02,6.08,10]dodec-11-ene-3,5-dione (II) Geometric parameters (Å, º)

**Table:**

Br1—C15	1.9004 (18)	C8—H8	1.0000
O1—C1	1.210 (2)	C8—C9	1.536 (3)
O2—C2	1.209 (2)	C9—H9	1.0000
N1—C1	1.393 (2)	C9—C10	1.504 (3)
N1—C2	1.397 (2)	C9—C11	1.508 (3)
N1—C12	1.432 (2)	C10—H10	1.0000
C1—C3	1.508 (3)	C10—C11	1.506 (3)
C2—C4	1.511 (2)	C11—H11A	0.9900
C3—H3	1.0000	C11—H11B	0.9900
C3—C4	1.539 (3)	C12—C13	1.384 (3)
C3—C8	1.564 (2)	C12—C17	1.383 (3)
C4—H4	1.0000	C13—H13	0.9500
C4—C5	1.557 (3)	C13—C14	1.388 (3)
C5—H5	1.0000	C14—H14	0.9500
C5—C6	1.499 (3)	C14—C15	1.379 (3)
C5—C10	1.533 (3)	C15—C16	1.379 (3)
C6—H6	0.9500	C16—H16	0.9500
C6—C7	1.324 (3)	C16—C17	1.391 (3)
C7—H7	0.9500	C17—H17	0.9500
C7—C8	1.503 (3)		
			
C1—N1—C2	112.82 (15)	C9—C8—H8	111.8
C1—N1—C12	122.65 (15)	C8—C9—H9	116.8
C2—N1—C12	124.12 (15)	C10—C9—C8	110.33 (16)
O1—C1—N1	123.98 (17)	C10—C9—H9	116.8
O1—C1—C3	127.52 (17)	C10—C9—C11	59.97 (14)
N1—C1—C3	108.50 (15)	C11—C9—C8	122.38 (18)
O2—C2—N1	123.97 (17)	C11—C9—H9	116.8
O2—C2—C4	127.59 (17)	C5—C10—H10	116.9
N1—C2—C4	108.43 (15)	C9—C10—C5	110.92 (16)
C1—C3—H3	109.6	C9—C10—H10	116.9
C1—C3—C4	105.22 (14)	C9—C10—C11	60.14 (14)
C1—C3—C8	113.26 (16)	C11—C10—C5	121.61 (18)
C4—C3—H3	109.6	C11—C10—H10	116.9
C4—C3—C8	109.59 (15)	C9—C11—H11A	117.8
C8—C3—H3	109.6	C9—C11—H11B	117.8
C2—C4—C3	104.86 (15)	C10—C11—C9	59.89 (13)
C2—C4—H4	109.9	C10—C11—H11A	117.8
C2—C4—C5	112.66 (15)	C10—C11—H11B	117.8
C3—C4—H4	109.9	H11A—C11—H11B	114.9
C3—C4—C5	109.55 (14)	C13—C12—N1	118.82 (16)
C5—C4—H4	109.9	C17—C12—N1	120.27 (16)
C4—C5—H5	111.8	C17—C12—C13	120.88 (17)
C6—C5—C4	106.45 (15)	C12—C13—H13	120.2
C6—C5—H5	111.8	C12—C13—C14	119.50 (18)
C6—C5—C10	110.37 (16)	C14—C13—H13	120.2
C10—C5—C4	104.30 (15)	C13—C14—H14	120.3
C10—C5—H5	111.8	C15—C14—C13	119.34 (18)
C5—C6—H6	122.4	C15—C14—H14	120.3
C7—C6—C5	115.13 (17)	C14—C15—Br1	118.97 (15)
C7—C6—H6	122.4	C16—C15—Br1	119.49 (15)
C6—C7—H7	122.7	C16—C15—C14	121.54 (18)
C6—C7—C8	114.59 (18)	C15—C16—H16	120.4
C8—C7—H7	122.7	C15—C16—C17	119.14 (18)
C3—C8—H8	111.8	C17—C16—H16	120.4
C7—C8—C3	107.32 (15)	C12—C17—C16	119.60 (18)
C7—C8—H8	111.8	C12—C17—H17	120.2
C7—C8—C9	110.18 (16)	C16—C17—H17	120.2
C9—C8—C3	103.63 (15)		

### 4-(4-Bromophenyl)-4-azatetracyclo[5.3.2.02,6.08,10]dodec-11-ene-3,5-dione (II) Hydrogen-bond geometry (Å, º)

*Cg*1 is the centroid of the C12–C17 ring.

**Table:**

*D*—H···*A*	*D*—H	H···*A*	*D*···*A*	*D*—H···*A*
C17—H17···O2^i^	0.95	2.40	3.175 (2)	139
C3—H3···*Cg*1^ii^	1.00	2.83	3.801 (2)	165

Symmetry codes: (i) *x*, *y*−1, *z*; (ii) −*x*+1/2, *y*+1/2, −*z*+3/2.
